# Cross-talk between circRNAs and m6A modifications in solid tumors

**DOI:** 10.1186/s12967-024-05500-4

**Published:** 2024-07-29

**Authors:** Fenfang Liu, Wendong Gu, Yingjie Shao

**Affiliations:** Department of Radiation Oncology, The Third Affiliated Hospital of Soochow University, 185 Juqian Street, Changzhou, 213003 China

**Keywords:** circRNAs, m6A, Cancer

## Abstract

Circular RNAs (circRNAs) possess unique biological properties and distribution characteristics that enable a variety of biological functions. N6-methyladenosine (m6A), a prevalent epigenetic modification in organisms, is regulated by factors including methyltransferases (writers), demethylases (erasers), and m6A-binding proteins (readers). These factors play critical roles in various pathophysiological processes. There is growing evidence that m6A modifications are common within circRNAs, affecting their synthesis, translation, translocation, degradation, and stability. Additionally, circRNAs regulate biological processes that influence m6A modifications. This review explores the metabolism and functions of m6A modifications and circRNAs, their interactions, and their specific regulatory mechanisms in different tumors, offering insights into m6A-circRNA interaction in cancer.

## Introduction

Circular RNAs (circRNAs) were identified within the genomes of viruses and the cytoplasm of eukaryotic cells as far back as the 1970s. Initially, they were dismissed as “junk” resulting from aberrant splicing events due to the limited understanding of that era [1–3]. Recent advancements in high-throughput RNA sequencing and bioinformatics have consistently demonstrated the existence of circRNAs across a wide range of organisms, including plants [4], parasites [5], and most mammals [6]. These circRNAs exhibit distinctive biological properties and distribution patterns, playing critical roles in regulating normal physiological functions and contributing to the development of various diseases [7].

N6-methyladenosine (m6A), the most prevalent form of mRNA modification, was discovered in the 1970s in the methylated nucleosides of mRNAs from Novikoff hepatoma cells [8, 9]. Advances in methods such as methylated RNA immunoprecipitation and sequencing (MeRIP-seq) have significantly enhanced the extensive study and understanding of m6A modification processes [10]. m6A represents a dynamically reversible epigenetic modification, primarily involving the methylation of the sixth nitrogen atom of adenine in mRNAs or non-coding RNAs (ncRNAs), facilitated by S-adenosylmethionine and regulated by specific m6A modification enzymes [11, 12]. This modification is predominantly found near termination codons, in 3′-untranslated regions (3′-UTRs), and within long internal exons [13, 14], playing roles in various pathological states and disease progressions.

Growing evidence suggests the widespread presence of m6A modifications within circRNAs and their interactions in diverse pathophysiological processes. Consequently, numerous studies have investigated the crosstalk between m6A modifications and circRNAs, aiming to understand their collaborative roles. This review discusses the biological metabolism and functions of circRNAs and m6A regulators, the interactions between circRNAs and m6A modifications, and the specific mechanisms of their interaction in different types of tumors. Additionally, it examines the potential and challenges of leveraging the interaction between circRNAs and m6A modifications in cancer therapy, based on current research findings.

## CircRNAs and M6A modifications

### CircRNAs

**Fig. 1:**
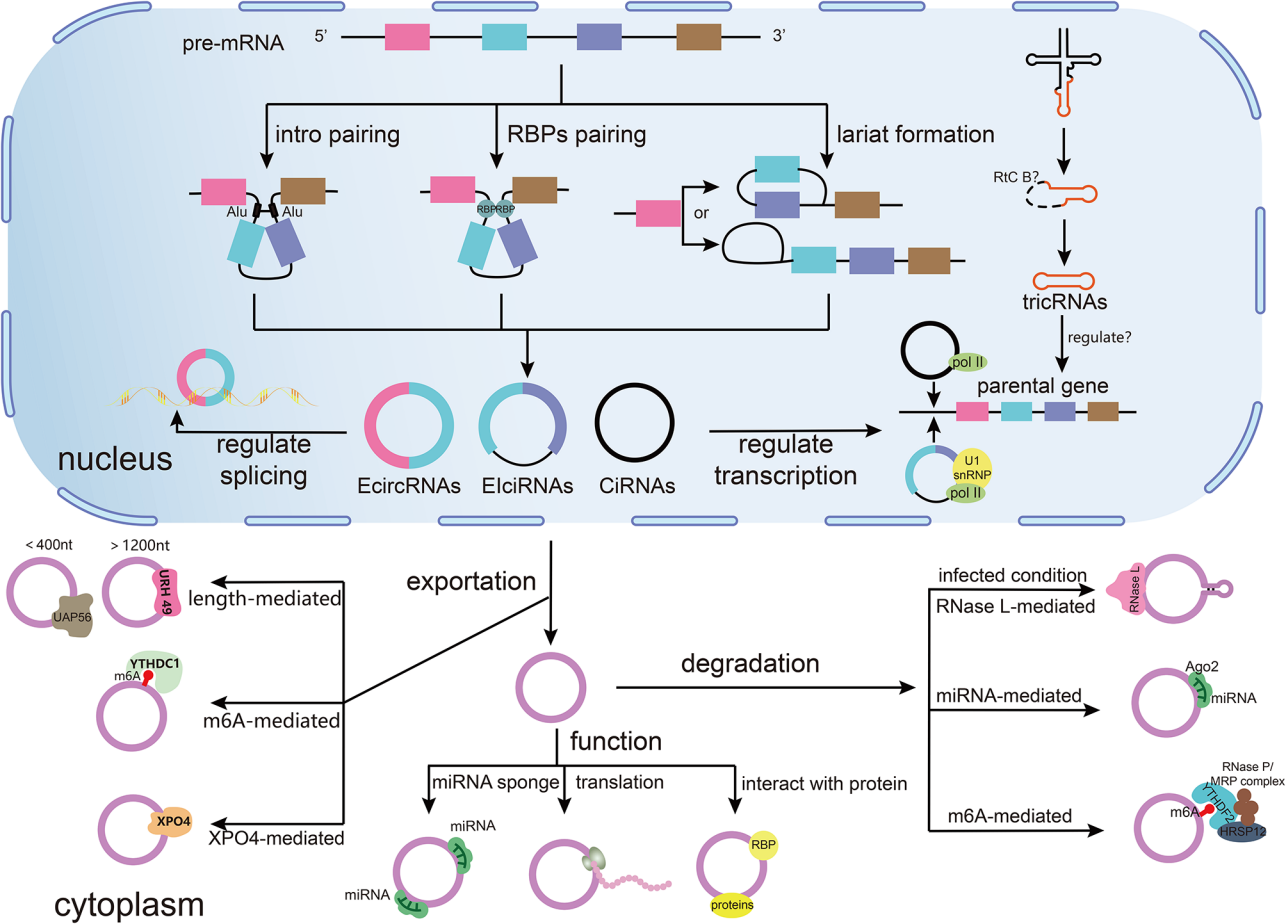
The biogenesis and function of circRNAs. CircRNAs can be synthesized in the nucleus by mechanisms such as intron pairing, RNA-binding protein (RBP) pairing, and lariat formation, where they are involved in regulating the transcription and splicing of parental genes, and partially translocated to the cytoplasm through length, m6A, and XPO4-mediated transport, where they are subjected to degradation by RNase L, miRNAs, and YTHDF2, and function by acting as miRNA sponges, translating, and interacting with proteins

circRNAs, characterized by their covalently closed loop structure without a 5′ cap and 3′ tail, are mainly synthesized through introns pairing [15], RNA binding proteins (RBPs) pairing [16], lariat formation [17] and other mechanisms. They are not only resistant to degradation by exonucleases such as exonucleases [18], but also form unique cell- and tissue-specific RNA loops via selective splicing [19]. CircRNAs can be categorized based on their splicing sequences into exonic circRNAs (EcircRNAs), exon-intron circRNAs (EIciRNAs), intronic circRNAs (CiRNAs), and others, such as tricRNAs which result from tRNA splicing and whose function is not yet clear [20–22]. Although primarily produced in the nucleus, circRNAs can move to the cytoplasm through various mechanisms [23]. For example, m6A-modified circNSUN2 can be exported from the nucleus to the cytoplasm by YTHDC1 in colorectal cancer, and circRNAs may undergo degradation by RNase L [24], miRNAs [25], and YTHDF2 [26], etc., under certain conditions. In addition, circRNAs in the nucleus possess the capability to regulate transcription and splicing of their parental genes through various mechanisms. For instance, Zhang et al. and Li et al. discovered that ciRNAs (ci-ankrd52) and EIciRNAs (circEIF3J/circPAIP2) in the nucleus can interact with Pol II and regulate the transcription of their parent genes [21, 27]. CircRNAs in the cytoplasm can influence post-transcriptional regulation [17]. They may serve as miRNA sponges, competing with miRNAs to bind to miRNA recognition elements on target genes and increasing target gene expression [28]. For example, circMETTL3 increase the expression of CDK1 by binding to miR-31-5p, thereby promoting the advancement of breast cancer [29]. Alternatively, they can be translated into polypeptides through mechanisms such as recruiting ribosomes in an internal ribosome entry site–dependent manner [30], or recruiting EIF4G2 and YTHDF3 in an m6A modification-dependent manner [31]. Additionally, circRNAs can interact with proteins, acting as protein sponges, decoys, scaffolds, or assisting in translocation to regulate the expression levels of themselves or downstream target molecules [18]. For example, circFoxo3 serves as a scaffold to mediate the combination of CDK2 and p21 to form a ternary complex, thereby blocking the cell cycle progression and keeping it in G1 phase [32] (Fig. 1).

### M6A modification regulators

Proteins involved in m6A modification include methyltransferases, demethylases, and RNA-binding proteins. Methyltransferases, also known as writers, catalyze N6-adenosine methylation and and regulate the biological functions of cells. Among them, METTL3 forms a stable complex with METTL14 and plays a key role as the core component of catalysis [33], while other members of the methyltransferase complex, such as WTAP [11], VIRMA/KIAA1429 [34], RBM15/15B [35], ZC3H13 [36], and HAKAI/CBLL1 [37], METTL16 [38], METTL5 [39], ZCCHC4 [40], are used as auxiliary subunits of the catalytic core, which affect the positioning, activity and stability of the methylation reaction. Demethylases, referred to as erasers, remove the m6A modification added by writers through complex mechanisms, thus maintaining a dynamic equilibrium of m6A modification. These enzymes include FTO [41], ALKBH5 [42] and ALKBH3 [43], which regulate the stability of mRNAs and affects cells proliferation. Readers, which are m6A RNA-binding proteins, specifically recognize and bind to N6-methyladenosine-modified RNAs, facilitating downstream biological functions [44]. Notably, YTHDF1/2/3 from the YT521-B homology (YTH) domain family are involved in mRNA translation and degradation [45–47]; YTHDC1/2, belonging to the YTH structural domain-containing proteins, are implicated in nuclear export, splicing, and mRNA translation [48, 49]; HNRNPA2B1, HNRNPC, and HNRNPG from the heterogeneous nuclear ribonucleoproteins (HNRNP) family enhance mRNA binding activity and regulate mRNA metabolism [50–52]. IGF2BP1/2/3 from the insulin-like growth factor 2 mRNA-binding proteins (IGF2BPs) family affect mRNA stability and translation [53]. EIF3, a eukaryotic translation initiation factor, binds directly to m6A-modified mRNAs (as m6A-induced ribosome entry sites) to initiate translation by recruiting the 43 S pre-initiation complex [54]. LRPPRC [55], Prrc2a [56], SND1 [57], and FMRP [58], newly identified m6A readers, function by binding to m6A-modified mRNAs (Table 1).

**Table 1:** Types and functions of m6A regulators

Type	Regulator	Role	Reference
Writers	METTL3	Methyltransferase catalyzes the active subunit	[33]
	METTL14	Recognition of substrates and enhancement of the catalytic activity of METTL3	[33]
	WTAP	Recruitment of METTL3-METTL14 heterodimers in nuclear spots	[11]
	VIRMA/KIAA1429	Inducing preferential methylation of regions near the 3′-UTR and termination codon of mRNAs	[34]
	RBM15/15B	Recruitment of m6A methyl complexes to specific sites of XIST	[35]
	ZC3H13	Linking and stabilizing WTAP and RBM15 for m6A deposition	[36]
	HAKAI/CBLL1	Composing MACOM and maintaining its stability	[37]
	METTL16	Catalyzing the methylation of U6 snRNA	[38]
	METTL5	Binding TRMT112 heterodimer to increase metabolic stability and promote 18 S rRNA methylation	[39]
	ZCCHC4	Catalyzing the methylation of 28 S rRNA	[40]
Erasers	FTO	Catalyzing the demethylation of m6A RNA, m6A_m mRNA, and m1A tRNA	[41]
	ALKBH5	Catalyzing the demethylation of m6A RNA	[42]
	ALKBH3	Catalyzing the demethylation of N6-meA tRNA	[43]
Readers	YTHDF1	Regulation of m6A mRNA translation and degradation	[45]
	YTHDF2	Mediating m6A RNA degradation and translation	[46]
	YTHDF3	Affecting the translation and degradation of m6A mRNAs	[47]
	YTHDC1	Regulating splicing of pre-mRNAs and mediating nuclear export of mRNAs	[48]
	YTHDC2	Dissociating the secondary structure of m6A CDS to improve translation efficiency	[49]
	HNRNPA2B1	Regulating the splicing of m6A RNAs	[50]
	HNRNPC/HNRNPG	Increasing the binding activity to mRNAs to influence abundance and selective splicing of target mRNAs	[51, 52]
	IGF2BP1/2/3	Regulation of mRNA stability and translation	[53]
	eIF3	Mediating the initiation of translation of m6A-modified 5′-UTR mRNAs in a cap-independent manner	[54]
	LRPPRC	Upregulating m6A PD-L1 mRNA expression in hepatocellular carcinoma to promote tumor progression and immune escape	[55]
	Prrc2a	Regulating Olig2 mRNA stability in an m6A-dependent manner to modulate OPC specificity	[56]
	SND1	A new m6A reader	[57]
	FMRP	Mediating nuclear export of m6A mRNAs or stabilizing EGFR mRNAs in an m6A-dependent manner to promote colorectal cancer progression	[58]

### Interaction of m6A modifications with circRNAs

**Fig. 2:**
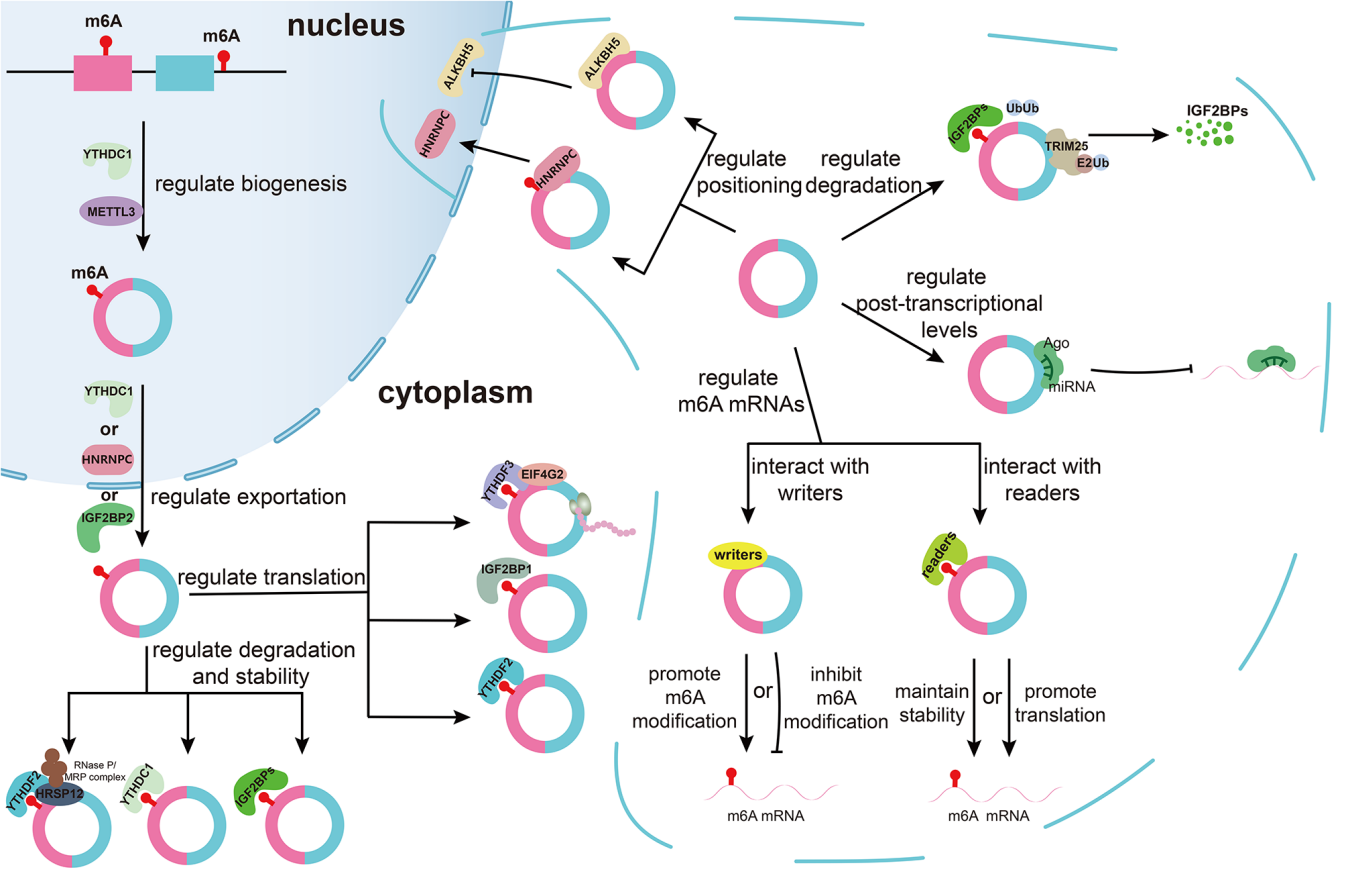
The crosstalk between m6A and circRNAs. m6A modifications affect the processes of biogenesis, translocation, translation, and degradation of circRNAs, which affect the post-transcriptional levels, translocation, and degradation of m6A regulators, as well as changes in the levels of m6A mRNAs

m6A modifications can act on circRNAs, influencing their metabolism and functions including biosynthesis, cellular distribution, translation, and degradation. Conversely, circRNAs can also affect the m6A modification process, underscoring a complex interaction between the two, as depicted in Fig. 2.

### M6A modifications regulate circRNAs

m6A modifications regulate the synthesis, degradation, translation, and cellular distribution of circRNAs, and affect tumorigenesis, progression and therapeutic resistance [59]. Changes in m6A modification levels can alter circRNA synthesis levels. Tang et al. observed that circRNA with increased levels in haploid male germ cells during spermatogenesis contains m6A modifications, which can be influenced by ALKBH5 and METTL3 [60]. Dattilo et al. found that YTHDC1 can cooperate with RNA helicase DDX5 to promote the synthesis of m6A-modified circRNAs in rhabdomyosarcoma (RMS), which control the proliferation activity of RMS cells [61]. Additionally, circRNAs can also be subjected to degradation by m6A modifications. For example, m6A-modified circRNAs can be cleaved by RNase P/MRP endoribonuclease in an HRSP12-dependent manner in the presence of YTHDF [26]. M6A-modified circMPP1 can be degraded in the presence of YTHDC1, which can be involved in regulating trophoblast function in the placental tissue [62]. CircRNAs, previously thought to lack translational capacity due to the absence of a 5′ translation initiation cap structure, have been found to be translatable in the presence of m6A. Yang et al. noted that m6A-modified circRNAs in human cells could initiate translation through EIF4G2 and the m6A reader YTHDF3 [31]. Duan et al. and Zhong et al. found that IGF2BP1 and YTHDF2 could bind specifically to circMAP3K4 and circMET, respectively, mediating their translation in a cap-independent manner, thereby contributing to the regulation of tumor progression [63, 64]. M6A modifications also play a role in the translocation of circRNAs, with Chen et al. showing that YTHDC1 can mediate the translocation of circNSUN2 from the nucleus to the cytoplasm by binding to its m6A modification site in colorectal cancer (CRC) [65]. Other researchers also found that IGF2BP2 and HNRNPC can mediate the transport of circCDK1 and circZBTB44 into the cytoplasm in laryngeal squamous cell carcinoma and renal cell carcinomas, respectively [66, 67].

### CircRNAs regulate m6A modifications

In addition to the relationships mentioned above, circRNAs also regulate the transport, degradation, and post-transcriptional levels of m6A regulators, and participate in tumor genesis, therapeutic resistance, metabolism and metastasis. CircRNAs can mediate the export of m6A regulators to the cytoplasm. Huang et al. found that circSTAG1, which is down-regulated in the the chronic unpredictable stress-treated mouse hippocampus and in peripheral blood of patients with major depressive disorder (MDD), can promote its translocation to the nucleus through decreased binding to ALKBH5. This leads to astrocyte dysfunction and depressive-like behavior [68]. CircRNAs also play a role in the degradation of m6A regulators. M6A-modified circNDUFB2 binds to IGF2BPs, serving as scaffolds to mediate the interaction between TRIM25 and IGF2BPs, ultimately promoting the ubiquitination and degradation of the IGF2BP and advancing NSCLC progression [69]. Similarly, circEZH2 prevents the ubiquitin-dependent degradation of IGF2BP2 by interacting with it and increases IGF2BP2 expression by binding to miR-133b, thereby promoting CRC progression by enhancing the stability of CREB1 mRNA [70]. Additionally, as “miRNA sponges,” circRNAs can regulate the post-transcriptional expression of m6A regulators. For instance, circMAP2K4 upregulates YTHDF1 expression by binding to has-miR-139-5p, promoting HCC progression [71]. Circ0072083 increases ALKBH5 expression by binding to miR-1252-5p, and reducing m6A modification, improving the stability of NANOG mRNA, and regulating temozolomide resistance in gliomas [72]. Circ_0072309 can increase FTO expression by binding to miR-607, which ultimately promotes NSCLC progression [73]. CircBACH2 increases HNRNPC expression by binding to hasmiR-944, which subsequently activates MAPK signaling pathway to promote the proliferation of breast cancer cells [74]. Finally, circRNAs can regulate the expression of m6A-modified mRNAs by interacting with m6A regulators. For example, circXPO1 can improve the stability of CTNNB1 mRNA by interacting with IGF2BP1, thus promoting the progression of LUAD [75]. CircUHRF2 can promote the interaction between IGF2BP1 and DDX27 mRNA by recruiting IGF2BP1, which inhibits the loss of DDX27 protein, and promotes the occurrence and metastasis of CRC [76].

## Crosstalk between M6A modifications and CircRNAs in solid tumors

Advanced research techniques have highlighted the role of circRNAs and m6A modifications in processes such as human cancer development. This review summarizes the crosstalk between circRNAs and m6A modifications across various tumors (Table 2 and 3).

**Table 2:** m6A modifications regulate circRNAs

Tumor type	circRNAs	m6A regulators	Effect on circRNAs	Role in cancer	Reference
Glioma	circDLC1	METTL3	Mediating m6A modification of circDLC1 to enhance its stability	Anti-oncogene	[77]
	circ_103239	METTL14	Mediating m6A modification of circRNA_103239 to enhance its expression	Anti-oncogene	[78]
	circMET	YTHDF2	Mediating translation of m6A circMET into MET404	Oncogene	[63]
NPC	circITCH	HNRNPC	Interacting with circITCH to reduce its expression	Anti-oncogene	[81]
HPSCC	circCUX1	METTL3	Mediating m6A modification of circCUX1 to enhance its stability	Oncogene	[83]
LSCC	circMMP9	IGF2BP2	Binding to m6A circMMP to enhance its stability	Oncogene	[84]
NSCLC	circIGF2BP3	METTL3/YTHDC1	Co-enhancing the expression of circIGF2BP3	Oncogene	[85]
	circFUT8	YTHDF2	Degrading m6A circFUT8	Oncogene	[86]
	circASK1	YTHDF2	Degrading m6A circASK1	Anti-oncogene	[87]
	circKRT17	METTL3	Mediating m6A modification of circKRT17 to enhance its stabilization	Oncogene	[88]
HCC	circARL3	METTL3/YTHDC1	Promoting circARL3 synthesis	Oncogene	[94]
	cFAM210A	RBM15/YTHDF2	Mediating m6A cFAM210A degradation	Anti-oncogene	[95]
	circSORE		Enhancing the stabilization of circSORE	Oncogene	[96]
	circMAP3K4	IGF2BP1	Mediating the translation of m6A circMAP3K4	Oncogene	[64]
	circHSP5	METTL3/YTHDC1	Mediating m6A circHSP5 translocation to the cytoplasm	Oncogene	[97]
	circKIAA1429	METTL3	Mediating m6A modification of circKIAA1429 to enhance its stability	Oncogene	[98]
	circMDK	IGF2BP1	Enhancing m6A circMDK stabilization	Oncogene	[99]
	circFUT8	METTL14/YTHDC1	Mediating m6A circFUT8 translocation to the cytoplasm	Oncogene	[100]
	circDLC1	KIAA1429	Negatively regulating circDLC1 expression	Anti-oncogene	[101]
	circ_0058493	METTL3/YTHDC1	Mediating m6A circ_0058493 translocation to the cytoplasm	Oncogene	[102]
	circCPSF6	ALKBH5/YTHDF2	Enhancing m6A circCPSF6 stabilization	Oncogene	[103]
	circSTX6	METTL14	Mediating m6A modification of circSTX6 to reduce its expression	Oncogene	[104]
	circCDYL	METTL3/YTHDC1	Promoting the synthesis of m6A circCDYL	Oncogene	[162]
CRC	circNSUN2	YTHDC1/IGF2BP1	Mediating circNSUN2 translocation to the cytoplasm	Oncogene	
	circ3823	YTHDF3/ALKBH5/YTHDF2	Mediating m6A circ3823 degradation	Oncogene	[111]
	circAFF2	ALKBH5/YTHDF2	Mediating m6A circAFF2 degradation	Anti-oncogene	[112]
	circ_0003215	YTHDF2	Mediating m6A circ_0003215 degradation	Anti-oncogene	[113]
	circFNDC3B	YTHDC1	Mediating circFNDC3B translocation to the cytoplasm	Anti-oncogene	[114]
	circYAP	YTHDF3	Mediating the translation of m6A circYAP	Oncogene	[115]
	circ1662	METTL3	Mediating m6A modification of circ1662 to enhance its expression	Oncogene	[116]
	circALG1	YTHDF1	Enhancing the role of m6A circALG1 and miR-342-5p	Oncogene	[117]
	circ_0000677	METTL3/YTHDF3	Enhancing the expression of circ_0000677	Oncogene	[118]
	circQSOX1	IGF2BP2/METTL3	Stabilizing circQSOX1	Oncogene	[119]
	circ_0124554	METTL3	Mediating m6A modification of circ_0124554 to enhance its expression	Oncogene	[120]
Breast cancer	circMETTL3	METTL3	Mediating m6A modification of circMETTL3 to enhance its expression	Oncogene	[29]
EC	circNAB1	ALKBH5/YTHDF2	Enhancing m6A circCPSF6 stabilization	Oncogene	[125]
Cervical cancer	circE7		Mediating circE7 translation	Oncogene	[128]
	circ_0000069		Stabilizing circ_0000069	Oncogene	[129]
	circCCDC134	ALKBH5/YTHDF2	Increasing m6A circCCDC134 stabilization	Oncogene	[130]
	circRNF13	METTL3/YTHDF2	Degrading m6A circRNF13	Oncogene	[131]
Ovarian cancer	circASXL1	METTL3/IGF2BP1	Increasing circASXL1 stabilization	Oncogene	[133]
	circNFIX	IGF2BPs	Increasing m6A circNFIX stabilization	Oncogene	[134]
	circPLPP4		Stabilizing circPLPP4	Oncogene	[135]
RCC	circPOLR2A	YTHDF2	Reducing m6A circPOLR2A expression	Oncogene	[137]
	circMET	YTHDC1	Mediating m6A circMET translocation to the cytoplasm	Oncogene	[138]
Bladder cancer	circSLC38A1		Increasing the expression of circSLC38A1	Oncogene	[141]
	circPSMA7	IGF2BP3	Stabilizing m6A circPSMA7	Oncogene	[142]
	circ_104797		Stabilizing circ_104797	Oncogene	[145]
Prostate cancer	circRBM33	METTL3	Mediating m6A modification of circRBM33 to enhance its expression	Oncogene	[146]
	circFAM126A	IGF2BP1	Binding m6A circFAM126A and enhancing its expression	Oncogene	[147]
	circDDIT4	METTL3/METTL14/WTAP	Promoting the synthesis of m6A circDDIT4	Anti-oncogene	[148]
Esophagus cancer	circRUNX1	IGF2BP2	Inhibiting m6A circRUNX1 degradation	Oncogene	[152]
Gastric cancer	circORC5	METTL14	Mediating m6A modification of circORC5 to reduce its expression	Oncogene	[153]
RMS	circVAMP3		Increasing the expression of circVAMP3	Oncogene	[156]
	circRNAs	YTHDC1	Cooperating with DDX5 to promote the synthesis of circRNAs	Oncogene	[61]
OS	circNRIP1	METTL3	Mediating m6A modification of circNRIP1 to enhance its expression	Oncogene	[157]
	circRNF220	METTL3	Mediating m6A modification of circRNF220 to enhance its expression	Oncogene	[158]
	circKEAP1	METTL3/METTL14/YTHDF2	Reducing m6A circKEAP1 stabilization	Anti-oncogene	[159]

NPC, Nasopharyngeal carcinoma; HPSCC, Hypopharyngeal squamous cell carcinoma; LSCC, Laryngeal squamous cell carcinoma; NSCLC, Non–small cell lung cancer; HCC, Hepatocellular carcinoma; CRC, Colorectal cancer; EC, Endometrial cancer; RCC, Renal cell carcinoma; RMS, Rhabdomyosarcoma; OS, osteosarcoma

**Table 3:** circRNAs affect m6A modification

Tumor type	circRNAs	m6A regulators	Effect on m6A regulators	Role in cancer	Reference
Glioma	circ_0072083	ALKBH5	Binding miR-1252-5p to upregulate ALKBH5	Oncogene	[72]
	circNEIL3	IGF2BP3	Stabilizing IGF2BP3	Oncogene	[79]
	circTTLL13	METTL3/YTHDF1	Recruitment of METTL3 and YTHDF1 to enhance m6A OLR1 mRNA stabilization	Oncogene	[80]
OSCC	circFOXK2	IGF2BP3	Interacting with IGF2BP3 and promoting m6A GLUT1 mRNA stabilization	Oncogene	[82]
LSCC	circCDK1	IGF2BP2	Competitive binding of IGF2BP2 to reduce m6A CPPED1 mRNA stabilization	Oncogene	[66]
NSCLC	circNDUFB2	IGF2BPs	Binding IGF2BPs and promoting their degradation	Anti-oncogene	[69]
	circ_0072309	FTO	Binding miR-607 to upregulate FTO	Oncogene	[73]
	circVMP1	METTL3	Binding miR-524-5p to upregulate METTL3	Oncogene	[89]
	circFBXW7	YTHDF3	Induction of β-catenin degradation to downregulate YTHDF3	Oncogene	[90]
	circXPO1	IGF2BP1	Binding IGF2BP1 to enhance CTNNB1 mRNA stabilization	Oncogene	[75]
	circNOTCH	METTL14	Competitive binding of METTL14 to maintain m6A NOTCH1 mRNA stabilization	Oncogene	[91]
	circEML4	ALKBH5	Promoting ALKBH5 translocation to the cytoplasm	Oncogene	[92]
HCC	circMAP2K4	YTHDF1	Binding hsa-miR-139-5p to upregulate YTHDF1	Oncogene	[71]
	circGPR137B	FTO	Binding miR-4739 to upregulate FTO	Anti-oncogene	[105]
	circCCAR1	WTAP	Binding miR-127-5p to upregulate WTAP	Oncogene	[106]
	circKIAA1429	YTHDF3	Maintaining the role of YTHDF3 in enhancing m6A Zeb1 mRNA stabilization	Oncogene	[107]
	circRERE	ZC3H13	Inhibition of the action of ZC3H13 on GBX2	Oncogene	[108]
	circMEG3	METTL3	Inhibition of METTL3-mediated m6A modification of Cbf5 mRNA	Anti-oncogene	[109]
	circRHBDD1	YTHDF1	Promoting the role of YTHDF1 in mediating m6A PIK3R1 mRNA translation	Oncogene	[110]
CRC	circEZH2	IGF2BP2	Inhibiting IGF2BP2 degradation and binding miR-133b to upregulate IGF2BP2	Oncogene	[70]
	circUHRF2	IGF2BP1	Recruiting IGF2BP1 and promoting its interaction with DDX27 mRNA	Oncogene	[76]
	circPTK2	YTHDF1	Binding miR-136-5p to upregulate YTHDF1	Oncogene	[121]
	circMYH9	HNRNPB2A1	Inhibition of HNRNPB2A1 binding to m6A p53 pre-mRNA and reduction of its stabilization	Oncogene	[122]
	circASPH	IGF2BP2	Binding and stabilizing IGF2BP2 to enhance m6A STING mRNA stabilization	Oncogene	[123]
Breast cancer	circMETTL3	METTL3	Binding miR-34c-3p to upregulate METTL3	Oncogene	[124]
	circBACH2	HNRNPC	Binging has-miR-944 to upregulate HNRNPC	Oncogene	[74]
EC	circCHD7	IGF2BP2	Interacting with IGF2BP2 to enhance the stability of PDGFRB mRNA	Oncogene	[126]
Cervical cancer	circARHGAP12	IGF2BP2	Binding IGF2BP2 to enhance m6A FOXM1 mRNA stabilization	Oncogene	[132]
Ovarian cancer	circRAB11FIP1	FTO	Direct binding FTO mRNA and enhancing its expression	Oncogene	[136]
RCC	circZBTB44	IGF2BP3	Interacting with IGF2BP3 to upregulate HK3	Oncogene	[67]
	circRARS	IGF2BP3	Binding IGF2BP3 to maintain the stabilization of m6A CAPN15/CD44	Oncogene	[139]
	circPPAP2B	HNRNPC	Binding HNRNPC to affect its degradation and facilitate its translocation to the nucleus	Oncogene	[140]
Bladder cancer	circPTPRA	IGF2BP1	Competitive binding of IGF2BP1 to reduce stabilization of m6A MYC/FSCN1 mRNAs	Anti-oncogene	[143]
	circ_0008399	WTAP	Interacting with WTAP to promote m6A modification of TNFAIP3 mRNA	Oncogene	[144]
Prostate cancer	circPDE5A	WTAP	Inhibition of WTAP-mediated modification of EIF3C mRNA m6A	Anti-oncogene	[149]
	circABCC4	IGF2BP2	Promoting the role of IGF2BP2 in enhancing CCAR1 mRNA stabilization	Oncogene	[150]
	circARHGAP29	IGF2BP2	Binding IGF2BP2 to maintain c-Myc mRNA stabilization	Oncogene	[151]
melanoma	circ_0053943	IGF2BP3	Binding IGF2BP2 to maintain EGFR mRNA stabilization	Oncogene	[155]
Osteosarcoma	circ_0000285	IGF2BP3	Binding miR-409-3p to upregulate IGF2BP3	Oncogene	[160]
	circCTNNB1	RBM15	Binding RBM15 to maintain m6A GPI/HK2/PGK1 stabilization	Oncogene	[161]

OSCC, Oral squamous cell carcinoma; LSCC, Laryngeal squamous cell carcinoma; NSCLC, Non–small cell lung cancer; HCC, Hepatocellular carcinoma; CRC, Colorectal cancer; EC, Endometrial cancer; RCC, Renal cell carcinoma; OS, osteosarcoma

### Glioma

m6A-modified circRNAs can function as competing endogenous RNAs (ceRNAs) in glioma tumorigenesis and proliferation. For instance, circDLC1 and circ_103239 increase their stability through m6A modification through m6A writers. Subsequently, circDLC1 promotes CTNNBIP1 transcription in vivo by competitively binding miR-671-5p, which inhibits glioma cells’ malignant proliferation [77]. Similarly, circ_103239 boosts MTSS1 expression by binding miR-182-5p, thereby inhibiting epithelial-to-mesenchymal transition (EMT) in glioma [78]. m6A-modified circRNAs also influence glioma progression through their translational effects. For example, m6A-modified circMET can be translated into MET404 in response to YTHDF2, activating MET receptors in the HGF/MET signaling pathway in glioma by interacting with the MET β-subunit. Consequently, combining MET404 antibodies with conventional MET inhibitors could offer a new therapeutic approach for GBM patients with MET hyperactivation [63].

CircRNAs play a critical role in regulating m6A modifiers in glioma tumorigenesis and drug resistance. CircNEIL3 interacts with IGF2BP3, protecting it from ubiquitination and degradation by E3 ubiquitin ligase-HECTD4. This activation of the YAP1 signalling pathway encourages macrophage infiltration into the tumor microenvironment in glioma cells, contributing to glioma progression [79]. Circ_0072083 and circTTLL13 specifically combat temozolomide resistance in gliomas. Mechanistically, circ_0072083 elevates ALKBH5 expression by binding to miR-1252-5p, which lessens m6A modification of NANOG mRNA and boosts its stability [72]. CircTTLL13 mediates the m6A modification of OLR1 pre-mRNA by recruiting METTL3 in the nucleus, enhancing m6A-modified OLR1 mRNA stability in the cytoplasm through YTHDF1 recruitment. This triggers the Wnt/β-catenin signaling pathway, influencing temozolomide resistance in glioma [80].

### Head and neck tumors

In nasopharyngeal carcinoma (NPC), Zhou et al. discovered that circITCH could impede NPC progression by diminishing the expression of downstream target molecules through binding to miR-224-3p. Conversely, HNRNPC in NPC cells could decrease its expression through circITCH interaction [81]. In oral squamous cell carcinoma (OSCC), Cui et al. identified that circFOXK2, abundantly present in OSCC cells, could enhance GLUT1 mRNA stability by interacting with IGF2BP3 in an m6A-dependent manner, thus speeding up aerobic glycolysis and OSCC progression [82]. In hypopharyngeal squamous carcinoma (HPSCC), Wu et al. noted that circCUX1, with increased expression in HPSCC tissues, could stabilize its expression by m6A modification due to METTL3. It could mitigate inflammatory factor release by binding to caspase 1, thereby promoting radiotherapy resistance in HPSCC [83]. In laryngeal squamous cell carcinoma (LSCC), Cao et al. observed that circMMP9, with elevated expression, could secure its expression through IGF2BP2 binding in an m6A-dependent manner and promote TRIM59 transcription by ETS1 recruitment, activating the PI3K/AKT signaling pathway and promoting LSCC progression [84]. Similarly, Li et al. reported increased circCDK1 synthesis in laryngeal cancer cells due to EIF4A3, which could bind IGF2BP2 and move to the cytoplasm, affecting CPPED1 mRNA stability, activating the PI3K-AKT pathway, and promoting LSCC cell proliferation and metastasis [66].

### Non–small cell lung cancer

Recent findings reveal that circIGF2BP3 binds YTHDC1 through METTL3-mediated m6A modification, enhancing its synthesis. It also mitigates its suppressive effect on PKP3 by sequestering miR-328-3p and miR-3173-5p, subsequently increasing the stability of OTUB1 mRNA. This process allows PKP3 to interact with FXR1, safeguarding PD-L1 from proteasome-mediated degradation, thereby fostering immunosuppression and immune escape in non–small cell lung cancer (NSCLC) [85]. Conversely, m6A-modified circFUT8, regulated by YTHDF2 can not only competitively interacte with YTHDF2 and blunt its binding to mFUT8, but also bind to miR-186-5p to enhance the stability of mFUT8, thereby promoting the malignancy in lung adenocarcinoma (LUAD) [86]. Moreover, m6A-modified circASK1, which is reduced in gefitinib-resistant LUAD due to YTHDF2-mediated degradation, can rescue the pro-apoptotic activity of ASK1/JNK/p38 signaling pathway through the interaction of its encoded protein ASK1-272a.a with Akt, thereby inducing apoptosis of LUAD cells and increasing gefitinib sensitivity [87]. CircKRT17, which gains stability through METTL3-mediated m6A modification in osimertinib-resistant LUAD, aids in the nuclear translocation and stabilization of YAP1 by recruiting EIF4A3. This action contributes to osimertinib resistance in LUAD [88].

CircRNAs are also implicated in lung cancer tumorigenesis and therapeutic resistance by influencing m6A regulators. Some circRNAs act by binding to microRNAs (miRNAs). For instance, circ_0072309 can upregulate FTO by binding to miR-607 [73]. CircVMP1 boosts METTL3 expression, leads to m6A modification of SOX2 mRNA, and enhances SOX2 mRNA stability by binding to miR-524-5p [89], which, in turn, promotes NSCLC cell proliferation and invasion. Other circRNAs function by being translated into peptides. For example, circFBXW7 engages with β-catenin in an m6A-dependent manner through its peptide circFBXW7-185, prompting the ubiquitination and degradation of β-catenin. This inhibits the activation of the classical Wnt signaling pathway, reduces Let-7d expression inhibition, lowers YTHDF3 levels, and curbs circFBXW7 translation, creating a feedback loop that regulates the properties and therapeutic resistance of LUAD stem cells [90]. Additionally, some circRNAs regulate downstream mRNAs. For instance, circXPO1 enhances LUAD progression by interacting with IGF2BP1 and increasing the stability of CTNNB1 mRNA [75]. CircNOTCH1 enhances NSCLC progression by competitively interacting with METTL14 and NOTCH1 mRNA, reducing the m6A modification level of NOTCH1 mRNA, thereby maintaining its stability [91]. Similarly, circEML4, through the delivery of M2-polarized macrophage extracellular vesicles induced by cigarette smoke extract, facilitates ALKBH5’s translocation to the cytoplasm in NSCLC cells. This action increases the m6A modification of SOCS2, lowers SOCS2 expression, stimulates the JAK-STAT pathway, and promotes NSCLC progression [92].

### Hepatocellular carcinoma

**Fig. 3:**
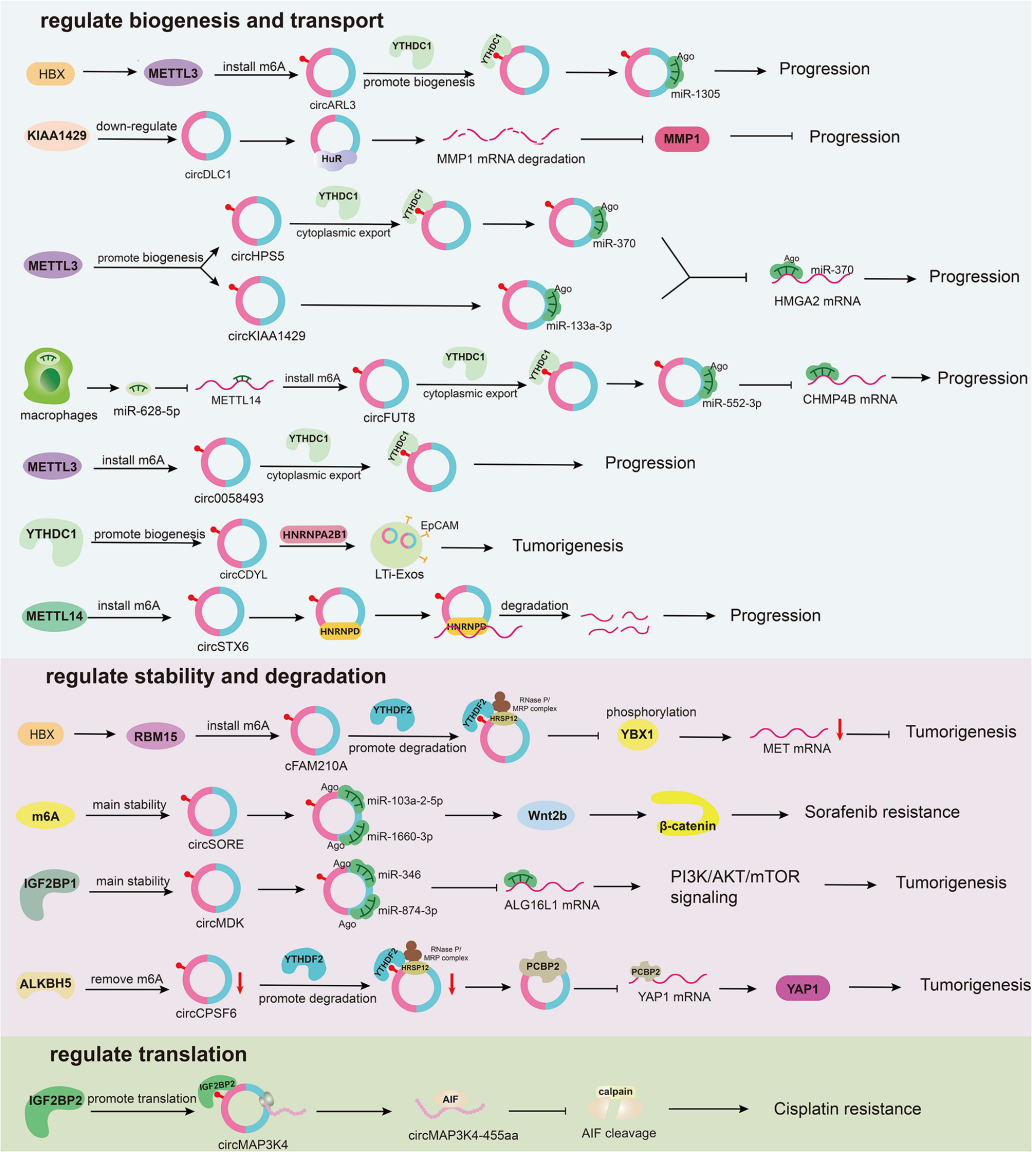
m6A modifications regulate circRNAs in hepatocellular carcinoma (HCC). M6A regulators mainly regulate the biogenesis, cytoplasmic export, translation, stability and degradation of circRNAs in HCC

Hepatocellular carcinoma, the most common type of primary liver cancer, is primarily caused by infections with hepatitis B virus (HBV) [93]. The HBx protein, produced following HBV infection, mediates m6A modification of circARL3 by increasing METTL3 expression. YTHDC1 then binds to the m6A-modified circARL3 and enhances its synthesis. This interaction enables circARL3 to bind miR-1305, facilitating the progression of HBV-related HCC [94]. Additionally, HBx can also mediate m6A modification of cFAM210A by inducing transcription of RBM15, and then degrade it through the YTHDF2-HRSP12-RNase P/MRP pathway to influence HCC progression [95]. CircRNAs play a role in HCC drug resistance; specifically, m6A-modified circSORE enhances HCC sorafenib resistance by activating the Wnt/β-catenin signaling pathway through binding to miR-103a-2-5p and miR-660-3p [96], m6A-modified circMAP3K4 inhibits cisplatin-induced cell death in HCC through its protein circMAP3K4-455aa encoded under the action of IGF2BP1, which inhibits AIF cleavage and nuclear distribution [64]. Furthermore, m6A-modified circRNAs contribute to HCC progression by binding miRNAs. For instance, circHSP5, modified by m6A and transported to the cytoplasm by YTHDC1 and METTL3, binds to miR-370. Similarly, m6A-modified circKIAA1429, upregulated by METTL3, binds to miR-133a-3p, and both target HMGA2 to facilitate HCC progression [97, 98]. Similarly, m6A-modified circMDK, stabilized by IGF2BP1, upregulates ATG16L1 expression and activates the PI3K/AKT/mTOR pathway by binding miR-346 and miR-874-3p [99]. The m6A-modified circFUT8, moved to the cytoplasm with METTL14’s help, promotes HCC by increasing CHMP4B expression through miR-552-3p binding [100]. In addition, various circRNAs, under the influence of m6A regulators can contribute to HCC development through different mechanisms. For instance, circDLC1, negatively regulated by KIAA1429, inhibits HCC proliferation by destabilizing MMP1 mRNA through binding HuR [101]. The m6A-modified circ_0058493, transported to the cytoplasm by YTHDC1, promotes HCC cells growth and metastasis [102]. CircCPSF6, with stability increased by ALKBH5 and YTHDF2, promotes HCC tumorigenesis by binding to PCBP2, preventing its interaction with YAP1 mRNA and thus maintaining YAP1 stability [103]. Decreased expression of circSTX6, mediated by METTL14, promotes ATF3 mRNA degradation by binding HNRNPD and promotes HCC progression [104] (Fig. 3).

**Fig. 4:**
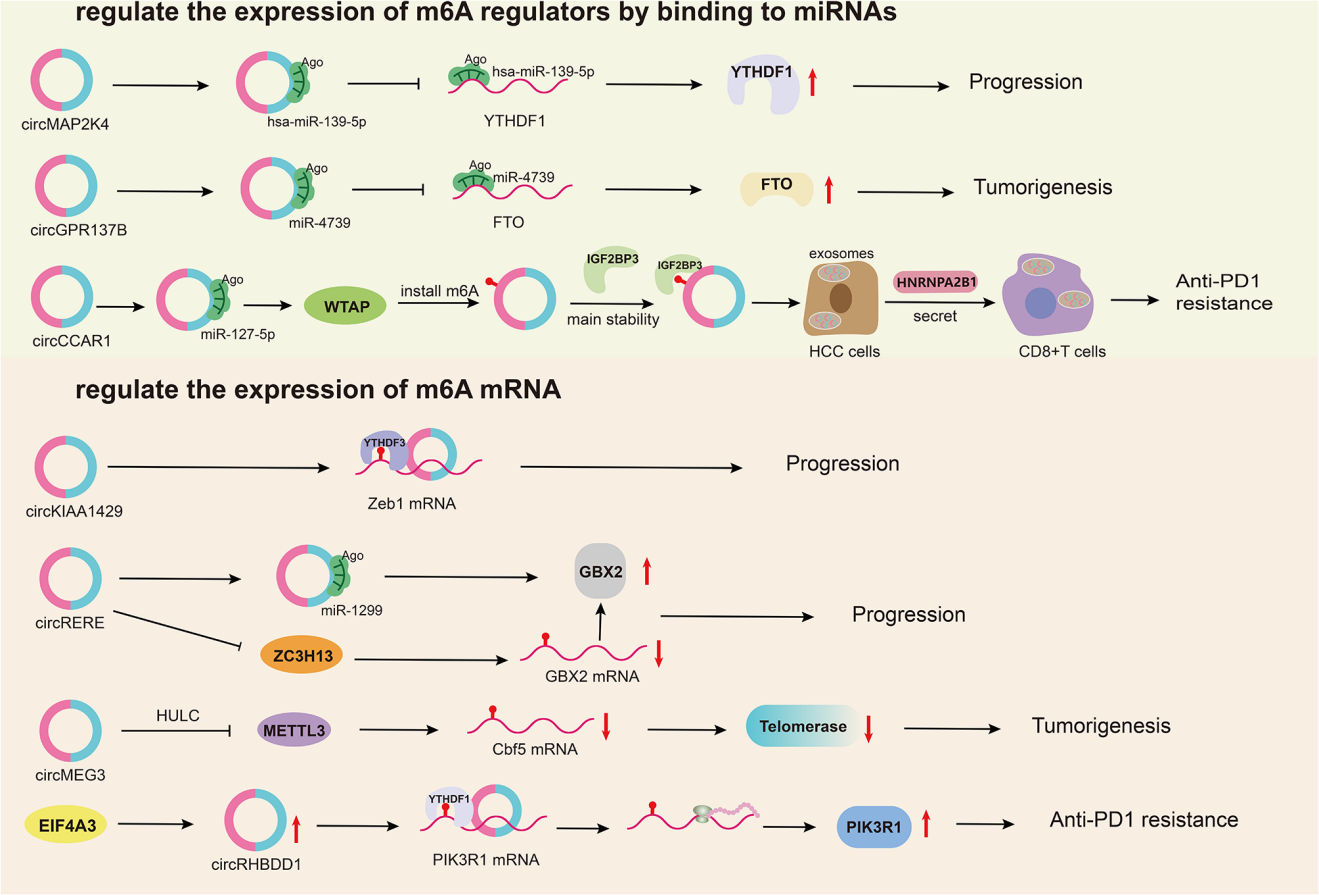
circRNAs regulate m6A modifications in hepatocellular carcinoma (HCC). CircRNAs mainly regulate the post-transcriptional levels of m6A regulators by binding to miRNAs in HCC. And regulate the expression of m6A mRNAs by interacting with m6A regulators in HCC

CircRNAs play a significant role in the development of HCC by influencing m6A regulators. They function as ceRNAs. For instance, circMAP2K4 facilitates HCC progression by enhancing YTHDF1 expression through hsa-miR-139-5p binding [71]. Similarly, circGPR137B boosts FTO expression through miR-4739 binding, which then regulates m6A modification and expression level of circGPR137B. This results in a positive feedback loop that helps inhibit HCC progression [105]. CircCCAR1 increases WTAP expression by interacting with miR-127-5p, and WTAP enhances circCCAR1’s stability through IGF2BP3 binding, forming a loop that induces resistance to anti-PD1 immunotherapy in HCC [106]. CircRNAs also regulate m6A-mRNA expression by acting on m6A regulators. For example, YTHDF3 increases the stability of m6A-modified Zeb1 mRNA, while circKIAA1429 acts on Zeb1 to promote the progression of HCC [107]. ZC3H13 reduces GBX2 expression by mediating its m6A modification, while circRERE upregulates GBX2 by miR-1299 binding, thereby promoting HCC [108]. circMEG3 inhibits the expression of Cbf5 through METTL3 dependent on HULC and inhibits telomerase activity, thereby preventing malignant differentiation in HCC stem cells [109]. YTHDF1-mediated translation of m6A-modified PIK3R1 mRNA is enhanced by increased circRHBDD1 synthesis, triggered by EIF4A3 interaction. This activates the PI3K/AKT pathway, accelerates glycolysis in HCC cells, and ultimately reduces HCC resistance to PD-1 immunotherapy [110] (Fig. 4).

### Colorectal cancer

M6A modifications play a critical role in the degradation of circRNAs in colorectal cancer (CRC). The m6A-modified circ3823, for instance, can be degraded by the YTHDF3/ALKBH5/YTHDF2 mechanism. However, circ3823 also influences the expression of TCF7 and its downstream molecules, MYC and CCND1, by binding to miR-30c-5p, which then activates the Wnt signalling pathway, fostering CRC progression [111]. Similarly, m6A-modified circAFF2 and circ_0003215 can also be degraded by YTHDF2, while circAFF2 inhibits the neddylation modification of CRC cells and enhances the radiosensitivity of CRC by facilitating the interaction of CAND1 with Cullin 1 [112]. The m6A-modified circ_0003215 elevates DLG4 expression by binding to miR-663b, promoting the k48-linked G6PD ubiquitination and inhibiting pentose phosphate metabolism in CRC cells, thereby inhibiting CRC progression [113]. Moreover, m6A modifications can mediate the cytoplasmic translocation of circRNAs. For instance, m6A-modified circNSUN2 can move to the cytoplasm under the influence of YTHDC1 and may promote liver metastasis in CRC by enhancing HMGA2 mRNA stability through the circNSUN2/IGF2BP2/HMGA2 RNA-protein ternary complex [65]. Another example is m6A-modified circFNDC3B, which, influenced by YTHDC1, can inhibit CRC progression and metastasis by binding to FXR2, maintaining RNF41 stabilization, and promoting the ubiquitination and degradation of ASB6 [114]. m6A modifications play a pivotal role in the translation of circRNAs. An example of this is m6A-modified circYAP, which is translated into YAP protein, YAP-220aa under the action of YTHDF3 and EIF4G2. Subsequently, YAP-220aa facilitates the nuclear translocation of YAP by interacting with LATS1. These mechanisms promote CRC invasiveness and liver metastasis [115]. Moreover, several circRNAs exhibiting increased stability and upregulated expression due to m6A modifications in CRC have been identified. These circRNAs include circ1662 that inhibits CRC epithelial mesenchymal transition (EMT) by binding to YAP1 protein, promoting its translocation to the nucleus and down-regulating SMAD3 expression [116]; circALG1 that promotes the metastasis of CRC by increasing PGF expression through binding to miR-342-5p [117]; circ_0000677 that facilitates CRC progression by increasing ABCC1 expression through binding to miR-655 [118]; circQSOX1 that promotes Treg cell-mediated CRC immune escape by increasing PGAM1 expression through binding to miR-326 and miR-330-5p [119]; and circ_0124554 that promotes CRC progression and radiotherapy resistance by increasing LASP1 expression through binding miR-1184 [120].

Additionally, numerous studies have highlighted how circRNAs regulate m6A regulators, with some blocking the action of miRNAs. For instance, circPTK2 can elevate YTHDF1 expression by binding to miR-136-5p, potentially enhancing CRC progression and chemoresistance by activating the Wnt/β-catenin signaling pathway [121]. CircEZH2 not only interacts with IGF2BP2, preventing its ubiquitin-dependent degradation, but also increases IGF2BP2 expression by binding to miR-133b, which enhances CREB1 mRNA stability and promotes CRC progression [70]. Some circRNAs can affect the expression of downstream target mRNAs through interactions with m6A regulators. For example, circMYH9 can recruit HNRNPB2A1 in the nucleus. This recruitment inhibits HNRNPB2A1’s binding to the m6A-modified p53 pre-mRNA, diminishing p53’s inhibitory effect on PHGDH expression, promoting serine-glycine metabolism and redox homeostasis, and ultimately facilitating CRC progression [122]. CircUHRF2, with expression upregulated by m6A modification in response to METTL3, enhances its interaction with DDX27 mRNA by recruiting IGF2BP1, promoting CRC occurrence and metastasis [76]. CircASPH enhances the stability of m6A-modified STING mRNA by binding and stabilizing the IGF2BP2 protein, which, in turn, promotes M2 macrophage polarization and accelerates CRC progression through exosome-mediated STING transfer to macrophages [123].

### Gynecological cancer

In breast cancer, Li et al. discovered that circMETTL3, with upregulated expression due to METTL3-induced m6A modification, could bind miR-31-5p and increase CDK1 expression, promoting breast cancer cels proliferation and metastasis [29]. By contrast, Ruan et al. found that circMETTL3 could boost METTL3 expression by binding to miR-34c-3p, hence suppressing triple-negative breast cancer cells proliferation and metastasis [124]. Additionally, Lv et al. found that circBACH2, with upregulated expression in breast cancer, could increase the expression of HNRNPC by binding to has-miR-944, activating the MAPK signaling pathway, and promoting breast cancer cells proliferation [74].

In endometrial cancer (EC), Zhang et al. found that circNAB1, which is upregulated by ALKBH5 in an m6A-YTHDF2-dependent manner, can enhance CDKN3 expression by binding to miR-876-3p, thus promoting the proliferation and progression of EC [125]. Similarly, Shi et al. discovered that circCHD7 enhances the stability of PDGFRB mRNA in an m6A-dependent manner by interacting with IGF2BP2, thereby activating the JAK/STAT signaling pathway and promoting the proliferation of EC cells [126].

In cervical cancer, Persistent infection with human papillomavirus (HPV) is a significant risk factor for cervical cancer progression [127]. Zhao et al. demonstrated that circE7, formed after HPV infection, can produce the E7 oncoprotein through its own m6A modification, contributing to cervical cancer cell growth [128]. Chen et al. discovered that circ_0000069, with increased stability due to m6A modification, promotes cervical cancer cell proliferation and metastasis by interacting with miR-4426 [129]. Further studies by Liang et al. and Shi et al. identified circCCDC134 and circRNF13, which express m6A modifications and are regulated by YTHDF2 in cervical cancer [130, 131]. Ji et al. found that circARHGAP12, with elevated m6A modification levels, can bind to m6A-modified FOXM1 mRNA through IGF2BP2. This binding leads to the formation of a circARHGAP12/IGF2BP2/FOXM1 triplex complex, enhancing FOXM1 mRNA stability and promoting cervical cancer cell proliferation and metastasis [132].

In ovarian cancer, circASXL1, stabilized by m6A modifications through METTL3 and IGF2BP1, increases RACGAP1 expression by binding to miR-320d. This activation of the PI3K/Akt pathway encourages the proliferation and metastasis of ovarian cancer [133]. The m6A-modified circNFIX, by binding to IGF2BPs, enhances IL-6R expression through miR-647 interaction. This activation of the JAK1/STAT3 pathway increases PD-L1 expression and stability, facilitating ovarian cancer cell proliferation and immune escape [134]. Li et al. discovered that circPLPP4, with increased stability from m6A modification, can induce cisplatin resistance in ovarian cancer by binding to miR-136 and upregulating PIK3R1 [135]. Additionally, circRAB11FIP1, upregulated in ovarian cancer, promotes autophagy and malignant progression by enhancing ATG5 and ATG7 mRNA modification through direct interaction with FTO mRNA and an increase in its expression, thereby promoting the malignant progression of epithelial ovarian cancer (EOC) [136].

### Urinary tumors

In renal cell carcinoma (RCC), circPOLR2A, regulated by YTHDF2 through m6A modification, enhances the interaction between PEBP1 and UBE3C. This interaction leads to the ubiquitination and degradation of PEBP1 by UBE3C, activating the ERK signaling pathway and promoting the progression and metastasis of clear cell renal cell carcinoma (ccRCC) [137]. The m6A-modified circMET, upregulated by NONO-TFE3 fusion proteins, interacts with CDKN2A mRNA in the cytoplasm, promoting its degradation and facilitating NONO-TFE3 tRCC progression [138]. Similarly, in RCC, m6A-modified circZBTB44 undergoes translocation into cells in response to HNRNPC to promote interaction with IGF2BP3, which, in turn, enhances macrophage M2 polarization by increasing HK3 expression and, ultimately, promotes immune escape from RCC [67]. CircRARS that bind to IGF2BP3 in ccRCC promote ccRCC progression by maintaining the stability of downstream m6A-modified target molecules, such as CAPN15, CD44, HMGA2, TNRC6A, and ZMIZ2 [139]. Lastly, upregulated m6A-modified circPPAP2B in ccRCC tissues stabilizes the HNRNPC/Vimentin/Importin α7 complex by binding to HNRNPC, influencing its ubiquitination and degradation. This process promotes the translocation of HNRNPC to the nucleus and facilitates the progression and metastasis of ccRCC [140].

In bladder cancer (BC), circSLC38A1, which is overexpressed due to m6A modification in BC cells, transcriptionally regulates TGF-β2 expression. This regulation occurs through the stabilization and increased expression of the ILF3 protein, thus facilitating BC progression and metastasis [141]. Similarly, m6A-modified circPSMA7 in BC cells, stabilized by IGF2BP3, upregulates MAPK1 mRNA expression by binding miR-128-3p, enhancing BC proliferation and metastasis [142]. Furthermore, circPTPRA binds competitively to IGF2BP1 in the cytoplasm, preventing it from recognizing downstream m6A-modified MYC and FSCN1 mRNAs. This action reduces the stability of these mRNAs and inhibits BC progression [143]. The increased production of circ_0008399, following the interaction between EIF4A and RBM3 pre-mRNA, interacts with WTAP in the nucleus. This interaction promotes the assembly of m6A methyltransferases (WTAP/METTL3/M3TTL14), leading to the m6A modification of TNFAIP3 mRNAs, which stabilizes them, thereby inhibiting BC cell apoptosis and promoting cisplatin resistance [144]. Circ_104797, with its stability enhanced by m6A modification, contributes to cisplatin resistance in BC cells by binding to miR-103a and miR-660-3p [145].

In prostate cancer (PCa), m6A-modified circRBM33 and circFAM126A are significantly upregulated and contribute to cancer proliferation and metastasis both in vivo and in vitro. Specifically, m6A-modified circRBM33, responding to METTL3, forms a binary complex with FMR1 that binds to PDHA1 mRNA. This complex increases PDHA1 mRNA stability and its translational output, enhancing oxidative phosphorylation (ox-pho) in PCa cells and promoting their growth and metastasis [146]. The m6A-modified circFAM126A, with increased transcriptional stability due to IGF2BP1 binding, upregulates CANX expression by binding to miR-505-3p. This modulation affects cholesterol synthesis and promotes the malignant progression of prostate cancer [147]. Additionally, circDDIT4, with increased synthesis due to m6A methyltransferase complex-mediated modification, acts as an RNA-binding protein sponge. It binds to ELAV1, reducing ANO7 expression and inhibiting PCa progression [148]. Moreover, circRNAs can regulate m6A regulators in PCa. For instance, circPDE5A, upregulated by the transcription factor FOXO4 and the RNA-binding protein eIF4A3, binds to WTAP, inhibiting EIF3C mRNA’s m6A modification and translation in a YTHDF1-dependent manner. This inhibition blocks the MAPK pathway activation, ultimately suppressing PCa progression and metastasis [149]. CircABCC4, upregulated by m6A modification in response to METTL3, enhances IGF2BP2 protein stability by promoting its interaction with CCAR1 mRNA. This enhancement activates the Wnt/β-catenin pathway, promoting PCa occurrence and metastasis [150]. Lastly, circARHGAP29, whose synthesis and cytoplasmic transport increase following EIF4A3 binding, interacts with IGF2BP2. This interaction stabilizes c-Myc mRNA, subsequently upregulating LDHA mRNA expression and promoting anaerobic glycolysis in PCa cells, which fosters PCa progression and resistance to docetaxel treatment [151].

### Other tumors

In esophageal cancer, Wang et al. identified that circRUNX1 expression was markedly increased in esophageal squamous carcinoma (ESCC) tissues. m6A-modified circRUNX1, by binding to IGF2BP2, could enhance its stability, elevate FOXP3 expression through binding to miR-449b-5p, and thus foster ESCC progression [152].

In gastric cancer, Fan et al. discovered that elevated circORC5 levels enhance gastric cancer progression by binding to miR-30c-2-3p and elevating EIF4B and AKT1S1 expression. METTL14 can reduce circORC5 levels through m6A modification, but it is poorly expressed in gastric cancer tissues, correlating with a poor prognosis [153]. Additionally, Zhang et al. identified an Epstein-Barr virus (EBV)-encoded circRNA, EBV-circRPMS1, highly expressed in EBV-associated gastric cancer (EBVaGC). It contributes to m6A regulator regulation by recruiting Sam68 to METTL3’s promoter region, thereby enhancing METTL3 expression and advancing EBVaGC progression [154].

In melanoma, Zhao et al. identified that circ_0053943, which is overexpressed in uveal melanoma (UM), stabilizes EGFR mRNA in an m6A-dependent manner by binding to IGF2BP3, activating the MAPK/ERK pathway and promoting the proliferation and aggressiveness of UM cells [155].

In rhabdomyosarcoma (RMS), Francesca et al. discovered that there is an upregulated expression of circVAMP3 in a representative cell line of alveolar rhabdomyosarcoma (RH4 cell line), and its m6A modification level is significantly different from its linear counterpart. Therefore, m6A modification may be involved in regulating the selective synthesis between the two. Moreover, circVAMP3 may regulate the cell cycle of RH4 through the AKT pathway and participate in its proliferation [156]. Interestingly, Dario et al. described the expression characteristics of circRNA in the context of RMS. Through genome-wide methods, they demonstrated that m6A reader YTHDC1 and RNA helicase DDX5 can jointly mediate the backsplicing of m6A-modified circRNAs in RMS, thereby promoting their synthesis. Then, the regulation of circRNAs expression by the two may be involved in the advancement of RMS [61].

In osteosarcoma, Meng et al. and Liu et al. have discovered that circNRIP1 and circRNF220 can be upregulated by m6A modification due to METTL3 in osteosarcoma cells. CircNRIP1 primarily functions through the miR-199a-FOXC2 pathway, while circRNF220 operates mainly through the miR-330-5p-survivin pathway [157, 158]. Additionally, Ji et al. found that circKEAP1 is destabilized by m6A modification in OS cells, promoting the ubiquitination and degradation of vimentin through the binding of its encoded protein, KEAP1-259aa, with the E3 ligase ARIH1. This process inhibits the malignant progression of OS cells [159]. Similarly, Long et al. and Yang et al. identified circ_0000285 and circCTNNB1 as actors on m6A regulators in OS cells. Circ_0000285 facilitates OS progression by enhancing IGF2BP3 expression through miR-409-3p binding [160]. By contrast, circCTNNB1 increases m6A modification of aerobic glycolytic genes, such as GPI, HK2, and PGK1, by binding to RBM15. This action stabilizes and boosts the expression of these target genes, promoting aerobic glycolysis and thus contributing to OS progression [161].

## Clinical application and prospects

The crosstalk between circRNAs and m6A modifications mainly affects the tumorigenesis, progression and treatment resistance of solid tumors. Among them, they can affect the above processes by participating in the proliferation, metastasis, metabolism, immunity and autophagy of tumor cells. Therefore, for these mechanisms of action, circRNAs and m6A modification regulators can serve as new tumor therapeutic targets. For example, Du et al. found that significantly upregulated circMDK in HCC is associated with m6A modification and poor survival in HCC patients, and it can enhance the expression of ATG16L1 by binding to miR-346 and miR-874-3, thereby activating the PI3K/AKT/mTOR signaling pathway and promoting the progression of HCC. According to this mechanism, poly (β-amino esters) (PAEs) were synthesized to mediate the delivery of circMDK siRNA (PAE-siRNA) in vivo, thus knocking down circMDK and achieving effective antitumor effects in animal models [99]. In addition, circRNAs and m6A modifier regulators may also provide novel strategies for therapeutic resistance of tumors. For example, Li et al. Showed that circFBXW7 effectively inhibits the abilities of LUAD stem cells and reverses resistance to Osimertinib by modulating Wnt pathway functions through the action of circFBXW7-185AA on β-catenin ubiquitination and inhibition. This mechanism provides new ideas and clinical research directions for the effect of circRNAs on TKIs therapeutic resistance in LUAD [90]. Finally, circRNAs and m6A modification regulators can also serve as biomarkers for novel tumor diagnosis. For example, Wei et al. reported that circCDYL can be loaded into exosomes under the action of HNRNPA2/B1 to form circCDYL-enriched epithelial cell adhesion molecule (EPCAM)-positive exosomes, namely liver tumor initiation exosomes (LTi-Exos). It may be combined with plasma alpha-fetoprotein (AFP) as a promising biomarker for early diagnosis of HCC [162].

In addition, according to the current relevant studies, there are still some problems to be solved about the cross-talk between circRNAs and m6A modifications: First, the differential expression of circRNAs and m6A modification regulators in different tumors causes different effects, but the reasons for the differences are still unclear. Second, methylated RNA immunoprecipitation and sequencing (MERIP-seq/ m6A-seq) is currently the main method for detecting m6A modification sites in circRNAs, but its specificity and sensitivity still need to be improved, for example, single nucleotide variation at methylation sites cannot be accurately detected [163]. Third, many studies have found that circRNAs and m6A modified regulators can serve as biomarkers for tumor therapy or diagnosis, but at present, it is still not possible to detect specific circRNAs in peripheral blood and achieve precise molecular therapy through siRNAs and other effects.

## Conclusions

In this review, we summarize the properties and functions of circRNAs and m6A modifications, and discuss their crosstalk effects in solid tumors. In different cancer types, m6A modification can be involved in regulating the synthesis, translation, transport, degradation and stability of circRNAs. Conversely, circRNAs can also regulate the post-transcriptional level and degradation of m6A modification regulators by binding miRNAs and interacting with m6A modification regulators, or regulate the expression of its downstream m6A-modified target mRNA. Their crosstalk provides a new perspective for the treatment and diagnosis of solid tumors.

## Data Availability

The data supporting the conclusion of this review have been included within the article.
